# Postoperative loss of skeletal muscle mass and visceral fat predicts survival and morbidity after surgically treated non-metastatic chondrosarcoma: a longitudinal CT morphometric study

**DOI:** 10.1186/s12957-026-04433-0

**Published:** 2026-05-28

**Authors:** Julian Kylies, Tobias M. Ballhause, Jana K. Striefler, Anna Duprée, Lennart Well, Karl- Heinz Frosch, Matthias Priemel

**Affiliations:** 1 Department of Trauma and Orthopedic Surgery, University Medical Center Hamburg-Eppendorf, Martinistraße 52, Hamburg, 20246 Germany; 2 Department of Oncology, Hematology and Bone Marrow Transplantation with Section Pneumology, Hubertus Wald University Cancer Center, University Medical Center Hamburg-Eppendorf, Hamburg, Germany; 3 Department of General, Visceral and Thoracic Surgery, University Medical Center Hamburg-Eppendorf, Hamburg, Germany; 4 Department of Diagnostic and Interventional Radiology and Nuclear Medicine, University Medical Center Hamburg-Eppendorf, Hamburg, Germany; 5 Department of Trauma Surgery, Orthopaedics and Sports Traumatology, BG Klinikum Hamburg, Hamburg, Germany

**Keywords:** Chondrosarcoma, Sarcopenia, CT morphometry, Skeletal muscle index, Visceral adipose tissue, Survival, Surgical outcomes, R0 resection, Longitudinal analysis

## Abstract

**Background:**

Chondrosarcoma is the second most common malignant primary bone tumor in adults. Surgical resection is the mainstay of treatment. However, outcomes remain heterogeneous even after complete (R0) resection within its oncologic margins. The prognostic relevance of CT-based body composition, both preoperatively and over the disease course, has not been defined in chondrosarcoma.

**Methods:**

A retrospective cohort study including 79 adults with histologically confirmed, non-metastatic (N0M0) chondrosarcoma treated by complete (R0) resection between 2010 and 2024 was conducted. Each patient had two evaluable CT examinations acquired on the same scanner, a preoperative baseline (tCT1) and a follow-up scan approximately one year postoperatively (tCT2; mean interval 13.3 ± 2.2 months). At the L3 level, skeletal muscle and visceral fat indices (Skeletal muscle index (SMI), Paraspinal Muscle Index (PSMI), Psoas Muscle Index (PMI), Skeletal Muscle Density (SMD), Visceral adipose Tissue (VAT)) were derived and compared over time. Changes were analyzed in relation to tumor localization and grade. Survival was assessed using Kaplan–Meier and Cox regression models. ROC analysis/Youden J Index identified prognostic thresholds (SMI loss ≥ 30%; VAT loss ≥ 25%). Preoperative sarcopenia was defined using established sex-specific SMI cutoffs.

**Results:**

A marked postoperative deterioration in SMI, PSMI, PMI, and VAT was observed, whereas muscle density (SMD) largely remained unchanged. Losses were most pronounced in trunk-localized tumors (e.g., SMI − 41.3 ± 20.4%) and in high-grade disease (SMI: G1 − 22.5 ± 13.2%, G2 − 36.3 ± 20.6%, G3 − 52.1 ± 15.6%). Patients with SMI loss ≥ 30% showed reduced median overall survival (64 vs. 116 months; *p* = 0.02), and VAT loss ≥ 25% was associated with similarly poor survival (42 vs. 88 months; *p* < 0.01). In multivariable analysis, both SMI loss ≥ 30% (HR 1.44, 95% CI 1.10–2.33; *p* = 0.02) and VAT loss ≥ 25% (HR 1.40, 95% CI 1.20–2.41; *p* = 0.01) independently predicted worse survival. Preoperative sarcopenia was associated with shorter median survival (42 vs. 116 months; *p* < 0.0001), higher surgical site infection rates (27% vs. 2%; *p* < 0.001), and prolonged hospitalization across anatomical sites (all *p* < 0.001), and remained an independent prognostic factor on adjusted analysis (HR 1.31, 95% CI 1.19–2.45; *p* < 0.01).

**Conclusions:**

In R0-resected, non-metastatic chondrosarcoma, both preoperative sarcopenia and postoperative declines in muscle and adipose tissue, particularly SMI loss ≥ 30% and VAT loss ≥ 25%, are independent predictors of reduced survival and worse postoperative outcomes. Leveraging routinely acquired staging CTs for morphometric analysis may enable early risk identification and guide personalized perioperative management.

## Introduction

Chondrosarcoma represents the second most common primary malignant bone tumor in adults and is characterized by marked heterogeneity in biological behavior and clinical outcomes [1–3]. Surgical resection remains the cornerstone of curative treatment, as chondrosarcomas are largely resistant to both chemotherapy and radiotherapy [4]. Despite advances in surgical technique and perioperative care, long-term prognosis remains highly variable and is predominantly determined by tumor grade, anatomical localization, and completeness of resection [5]. Even after complete (R0) resection, a considerable proportion of patients experience functional decline and reduced overall survival, underscoring the multifactorial nature of outcome determinants in this patient population.

Over the past decade, increasing attention has shifted from purely tumor-centered characteristics toward patient-related factors that reflect physiological reserve and tolerance of treatment [6–9]. Among these, sarcopenia, characterized by reduced skeletal muscle quantity and impaired muscle composition, has emerged as a key determinant of outcome. Across a range of malignancies, low muscle mass has been linked to delayed postoperative recovery, higher rates of complications, and inferior survival, underscoring the importance of host fitness in oncologic care [10]. Routine staging CT scans can be repurposed for quantitative body-composition profiling. By analyzing a single slice at the level of the third lumbar vertebra (L3), it is possible to derive standardized measures of global muscle mass and visceral fat compartments. Such CT-based morphometric indices can be obtained without additional imaging or radiation exposure and offer an objective surrogate of frailty and metabolic reserve [7, 11].

While sarcopenia has been shown to influence oncologic outcomes in myeloma, gastrointestinal, and lung cancer, its prognostic relevance in chondrosarcoma remains poorly understood [9, 12, 13]. In particular, it is unknown how muscle and adipose tissue evolve after surgical resection, whether these changes differ by tumor grade or anatomical site, and to what extent baseline sarcopenia or postoperative morphometric decline inform survival, functional recovery, or complication risk in this population.

Therefore, the present study aimed to perform a comprehensive CT morphometric analysis of surgically treated, non-metastatic (N0M0) chondrosarcoma patients with long-term follow-up. Specifically, we sought to quantify longitudinal changes in skeletal muscle and adipose tissue after complete (R0) resection, examine the influence of tumor localization and histological grade on the extent of morphometric decline, and evaluate the prognostic impact of preoperative and postoperative sarcopenia on survival, functional outcomes, and postoperative morbidity.

This study provides a detailed characterization of body composition dynamics in chondrosarcoma and identifies CT-based morphometric parameters as clinically relevant markers for risk stratification and postoperative recovery assessment in surgically treated patients.

## Materials and methods

### Study design and patient cohort

The study protocol received approval from the local institutional ethics board (ID: 2025-300576-WF) and adhered to the ethical principles of the Declaration of Helsinki. Owing to the retrospective and pseudonymized data structure, written informed consent was not required.

All patients treated for chondrosarcoma at the University Medical Center Hamburg-Eppendorf between 2010 and 2024 were screened (*n* = 107). Eligibility was confirmed after applying predefined selection criteria, resulting in a final cohort of 79 individuals. Only patients who had undergone complete resection of a primary chondrosarcoma and who demonstrated a TNM status of N0M0 at diagnosis and throughout follow-up were considered. For inclusion, two evaluable CT examinations were mandatory: a preoperative scan (tCT1) and a follow-up scan (tCT2) obtained at least 12 months after surgery as part of routine oncologic surveillance. Availability of essential clinical variables—tumor grade, anatomic location, resection margin, postoperative course including surgical site infection (SSI), hospital stay, and survival—was also required.

Patients were excluded when postoperative adjuvant chemotherapy or radiotherapy had been administered, when recurrent or metastatic disease was present at baseline, or when fewer than two suitable CT studies existed. Additional exclusion criteria comprised inadequate CT quality (motion artifacts, incomplete depiction of L3), previous spinal procedures, neuromuscular disorders, or synchronous malignancies potentially influencing body-composition metrics.

### Basic demographic data

**Fig. 1:**
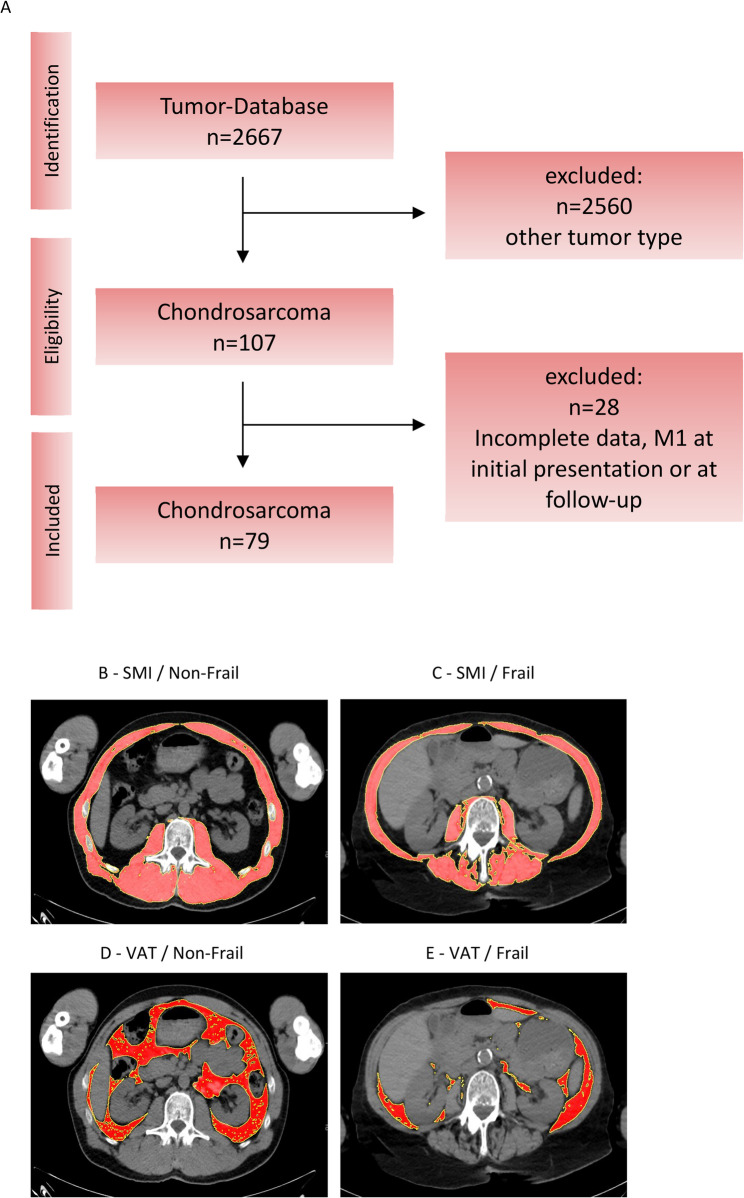
Study Design and CT Morphometric Assessment of Muscle and Visceral Adipose Tissue over the disease course. **A** Study design and patient selection. Skeletal muscle index (SMI) and visceral adipose tissue (VAT) were measured at baseline (tCT1) and follow-up (tCT2), revealing a progressive decline in both SMI (**B**, **C**) and VAT (**D**, **E**) during the observation period, shown are representative images of one patient

From our institutional sarcoma database, 2,667 patients treated for bone or soft-tissue sarcomas between 2010 and 2024 were identified. After excluding all cases without a diagnosis of chondrosarcoma (*n* = 2,560), 107 patients remained eligible for preliminary review. Subsequent exclusion of individuals lacking evaluable CT imaging, presenting with insufficient clinical documentation, or showing metastatic spread at diagnosis reduced the final study cohort to 79 patients (Fig. 1A).

The analyzed population consisted of 40 women (50.6%) and 39 men (49.4%), with a mean age of 55.1 ± 17.2 years at the time of diagnosis. Tumors of all histologic grades (G1–G3) and anatomical sites—including upper and lower extremities as well as axial locations—were represented within the cohort. Detailed demographic and clinical characteristics are presented in Table 1.

**Table 1:** Patient characteristics at baseline

Total (*n*)	Total	Male	Female
	79	39 (49.4%)	40 (50.6%)
Mean age (years)	55.1 ± 17.2	55.4 ± 17.6	54.9 ± 17.1
Mean follow up (months)	13.3 ± 2.2	13.3 ± 2.2	13.2 ± 2.3
Grading			
G1	27 (34.2%)	13 (16.5%)	14 (17.7)
G2	25 (31.6%)	10 (12.7%)	15 (18.9%)
G3	27 (34.2%)	15 (18.9%)	12 (15.3%)
ECOG at baseline (median/range)	1.0 (0.8)	1.0 (0.7)	1.0 (0.9)
Localization			
Trunk	33 (41.8%)	16 (20.3%)	17 (21.5%)
Lower Extremities	31 (39.2%)	16 (20.3%)	15 (18.9%)
Upper Extremities	15 (19.0%)	8 (10.1%)	7 (8.8%)

Illustrated are the baseline characteristics of the patient cohort

For each patient, two CT examinations were available for morphometric analysis. The median time span between the preoperative CT (tCT1) and the follow-up scan (tCT2) was 13.3 ± 2.2 months, providing an adequate interval to capture postoperative alterations in body composition. The initial CT was typically performed shortly before surgical management (mean 0.3 ± 0.2 months). The second scan was obtained approximately one year after surgery (mean 12.5 ± 2.2 months) as part of standardized surveillance protocols for chondrosarcoma follow-up. While morphometric changes were assessed between the two CT examinations obtained approximately one year apart, overall survival was evaluated over the entire available clinical follow-up period after surgery, which extended well beyond the imaging interval.

### CT acquisition and body-composition assessment

All CT examinations were performed on the same scanner platform (Siemens SOMATOM Force, Siemens Healthineers, Erlangen, Germany) using a standardized institutional protocol (100/150 kV with tin filtration; pitch 0.5; collimation 0.6 mm; acquisition slice thickness 1 mm, reconstructed to 5 mm). All CT examinations were performed according to the standardized institutional contrast-enhanced CT protocol of our radiology department, which includes intravenous administration of 80 mL Iomeprol (Imeron 350 M, Bracco Imaging, Milan, Italy) with image acquisition 80 s after injection, corresponding to the venous contrast phase. Quantitative morphometry was carried out using Fiji (Version 2.3.0/1.53q). For each dataset, a single axial slice centered at the mid-height of the third lumbar vertebra was analyzed. Muscle compartments were delineated manually, followed by semi-automated filtering of voxels within muscle-typical attenuation ranges to generate cross-sectional areas (–29 to + 150 HU). Cross-sectional areas were normalized to height squared to derive the skeletal muscle index (SMI), paraspinal muscle index (PSMI), and psoas muscle index (PMI). Patient height was obtained retrospectively from the electronic medical records, where it is routinely documented as part of the standard preoperative clinical assessment. Muscle density (SMD) was calculated as the mean attenuation within the paraspinal muscle ROI.

Visceral adipose tissue (VAT) was quantified on the same axial slice using attenuation limits of − 190 to − 30 HU. Manual tracing of the abdominal cavity ensured exclusion of subcutaneous fat [7, 11].

Clinical and demographic parameters were extracted from electronic health records at both imaging time points.

### Statistical analysis

All statistical procedures were conducted using SPSS Statistics v29 (IBM Corp., Armonk, NY, USA), and visualizations were generated with GraphPad Prism v10.2.2 (GraphPad Software, La Jolla, CA, USA). Continuous variables were summarized as mean ± standard deviation. Group comparisons were performed with Mann–Whitney U tests (two groups) or Kruskal–Wallis tests (three or more groups). A p-value < 0.05 was considered statistically significant.

To determine morphometric thresholds predictive of survival, receiver operating characteristic (ROC) analyses were performed. Using ROC analysis and Youden’s Index, exploratory thresholds of ≥ 30% SMI decline and ≥ 25% VAT decrease were identified as the most discriminatory values for survival within the study cohort.

Overall survival (OS) was defined as the interval between definitive surgery and death from any cause. Kaplan–Meier analysis was used to estimate survival probabilities, stratified by baseline sarcopenia status as well as by presence of SMI or VAT decline beyond the identified thresholds. Log-rank testing assessed differences between survival curves.

The independent prognostic value of body-composition changes was evaluated using multivariable Cox proportional hazards models. Adjusted covariates included age, sex, ECOG performance status, tumor grade, tumor size, anatomic site, and margin status. Hazard ratios (HRs) with corresponding 95% confidence intervals (CIs) were calculated, and ties were resolved using the Efron method. Confidence intervals were derived directly from the regression coefficients and their corresponding standard errors within the Cox proportional hazards models. The included covariates demonstrated stable coefficient estimation without evidence of marked variance inflation or implausible hazard ratio instability. Covariates included in the multivariable models were pre-specified based on established prognostic factors in sarcoma and supported by the univariate analyses. The proportional hazards assumption was formally assessed using time-dependent covariate testing in SPSS and no significant violations were observed. The proportional hazards assumption was formally assessed using time-dependent covariate testing in SPSS and no significant violations were observed. Additional graphical assessments such as Schoenfeld residual analyses were not performed.

## Results

### CT morphometric decline in male and female chondrosarcoma patients following surgical resection

Across the cohort of patients with chondrosarcoma, clear longitudinal changes in body composition were evident when comparing preoperative (tCT1) and postoperative (tCT2) imaging. The evolution of morphometric parameters consistently pointed toward a progressive reduction in skeletal muscle mass and visceral adiposity over time.

**Fig. 2:**
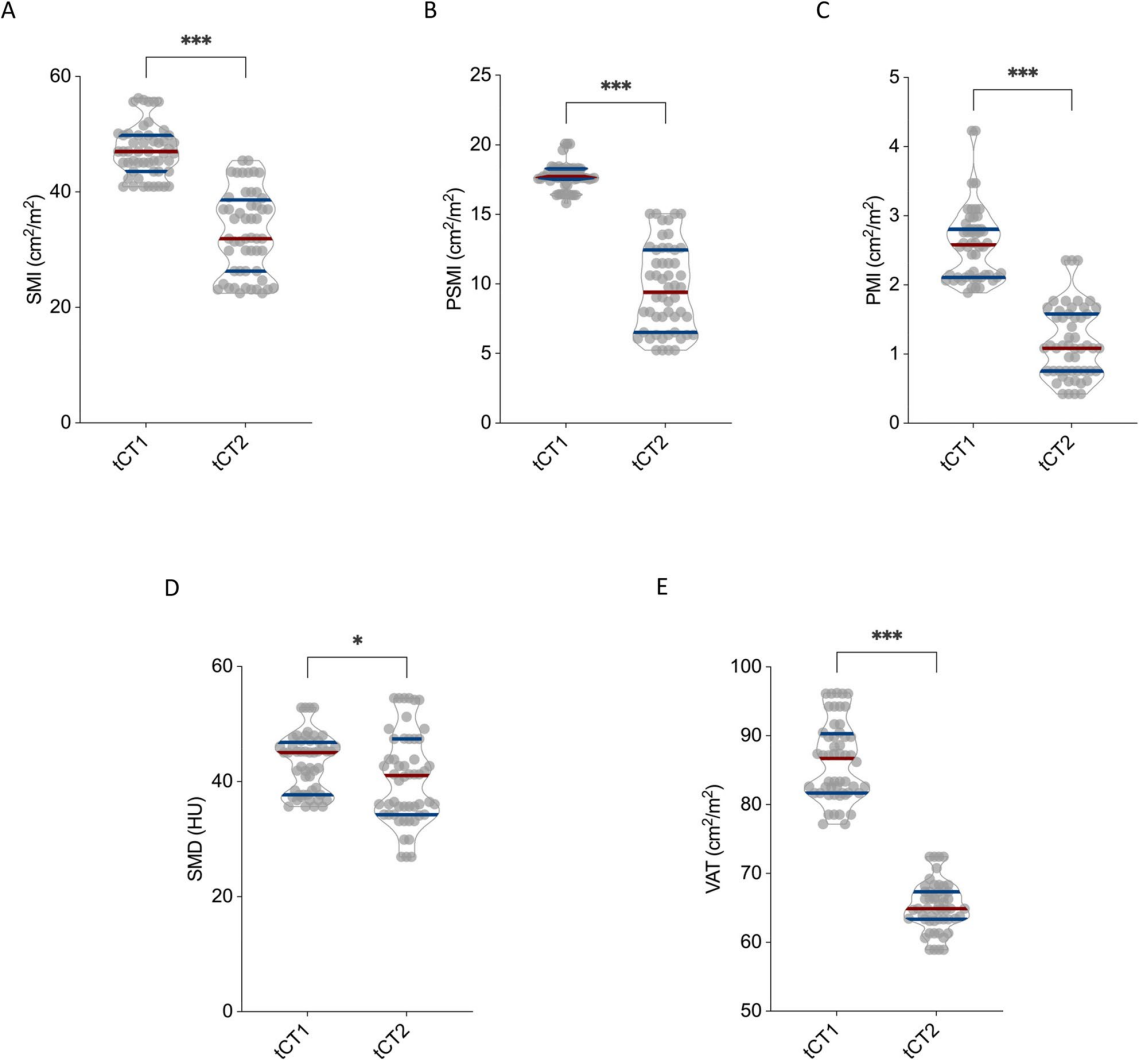
CT Morphometric Decline in Male Chondrosarcoma Patients Following Surgical Resection. Depicted are the longitudinal changes in skeletal muscle mass, skeletal muscle density (SMD), and visceral adipose tissue (VAT) across the male cohort of chondorsarcoma patients between tCT1 and tCT2. Skeletal muscle index (SMI) (**A**), paraspinal muscle index (PSMI) (**B**), psoas muscle index (PMI) (**C**), SMD (**D**) and VAT (**E**) all showed significant declines over the disease course. *p < 0.05, **p < 0.01 ***p < 0.001, ****p < 0.0001

Men demonstrated substantial declines across all muscle-related indices. Mean SMI dropped from 47.1 ± 4.4 cm²/m² at tCT1 to 32.8 ± 7.3 cm²/m² at tCT2 (*p* < 0.001; Fig. 2A). Correspondingly, PSMI fell from 17.8 ± 0.9 cm²/m² to 9.7 ± 3.1 cm²/m² (*p* < 0.001; Fig. 2B), and PMI was reduced from 2.6 ± 0.5 cm²/m² to 1.2 ± 0.5 cm²/m² (*p* < 0.001; Fig. 2C). In men, muscle density exhibited a modest decrease, with SMD declining from 43.2 ± 5.0 HU to 40.5 ± 7.8 HU (*p* = 0.03; Fig. 2D). VAT also showed a marked reduction, decreasing from 86.3 ± 5.6 cm² to 65.2 ± 3.5 cm² (*p* < 0.001; Fig. 2E).

**Fig. 3:**
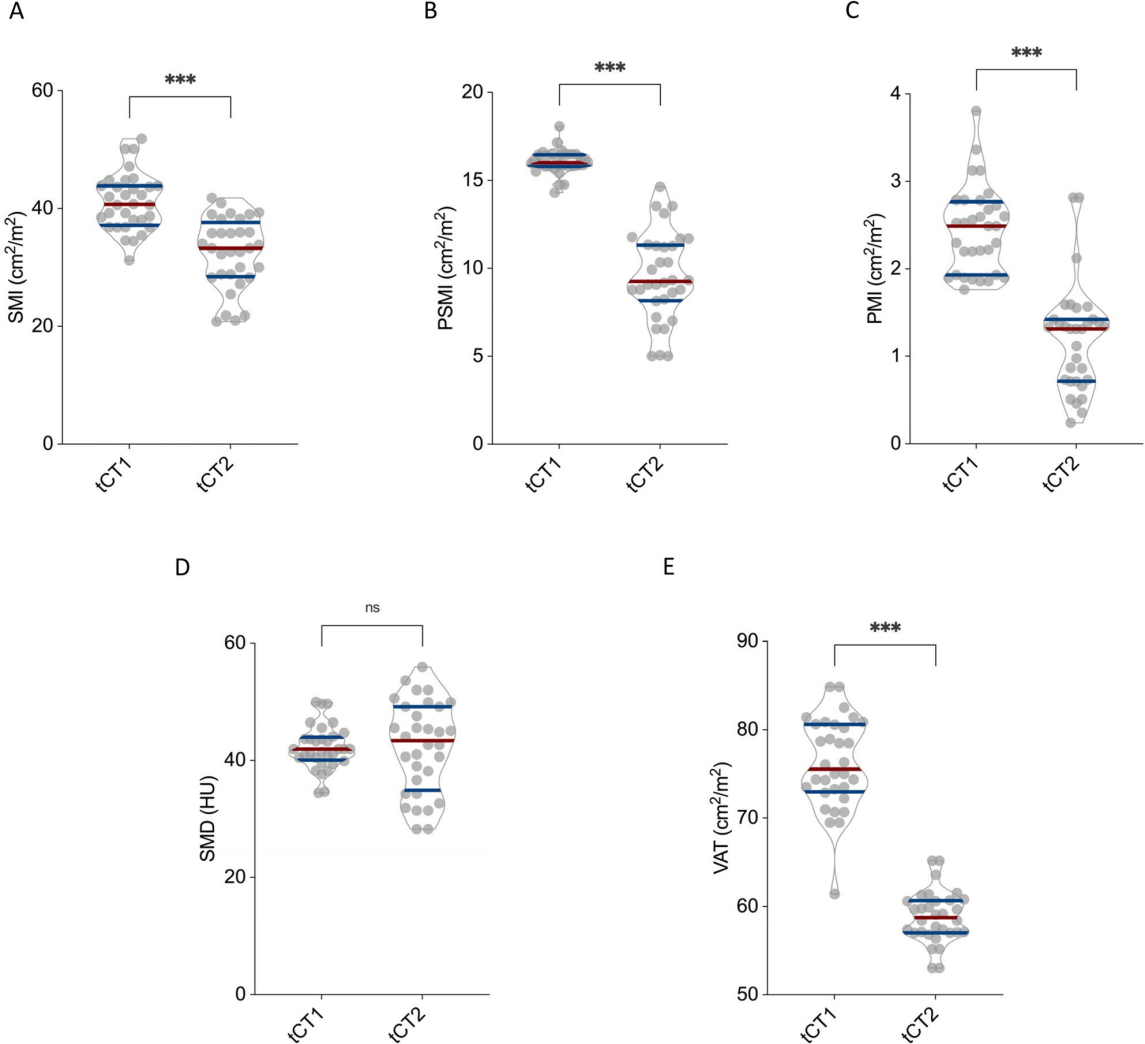
CT Morphometric Decline in Female Chondrosarcoma Patients Following Surgical Resection. Depicted are the longitudinal changes in skeletal muscle mass, skeletal muscle density (SMD), and visceral adipose tissue (VAT) across the female cohort of chondrosarcoma patients between tCT1 and tCT2. Skeletal muscle index (SMI) (**A**), paraspinal muscle index (PSMI) (**B**), psoas muscle index (PMI) (**C**), and VAT (**E**) all showed significant declines over the disease course, whereas SMD (**D**) remained stable without significant changes. *p < 0.05, **p < 0.01 ***p < 0.001, ****p < 0.0001

Women showed a comparable pattern of postoperative morphometric decline. SMI decreased from 41.1 ± 4.8 cm²/m² to 32.5 ± 6.0 cm²/m² (*p* < 0.001; Fig. 3A), and reductions in PSMI (16.1 ± 0.7 to 9.5 ± 2.5 cm²/m²; *p* < 0.001; Fig. 3B) and PMI (2.4 ± 0.5 to 1.2 ± 0.6 cm²/m²; *p* < 0.001; Fig. 3C) mirrored those observed in male patients. Unlike the male subgroup, however, SMD remained unchanged in women (42.2 ± 3.8 HU vs. 42.3 ± 7.8 HU; *p* = 0.33; Fig. 3D). VAT significantly declined from 76.1 ± 5.2 cm² to 58.8 ± 2.9 cm² (*p* < 0.001; Fig. 3E).

Overall, both male and female patients experienced substantial postoperative declines in muscle mass and visceral fat. Although reductions in SMI, PSMI, PMI, and VAT were consistently observed across sexes, muscle quality—as assessed by SMD—remained largely stable, particularly among women. These results indicate a pronounced postoperative sarcopenic trajectory in chondrosarcoma patients.

### Morphometric decline of muscle and visceral fat is most pronounced in trunk-localized chondrosarcomas

After establishing a consistent decline in muscle and adipose tissue in both male and female patients, we next explored whether the extent of morphometric deterioration differed by tumor localization. To enable standardized comparison across anatomical sites, relative percentage changes were analyzed, and male and female patients were pooled for subsequent analyses.

Of the 79 surgically treated chondrosarcoma patients included in this study, 33 (41.8%) had tumors localized to the trunk (including pelvis and thoracic wall), 31 (39.2%) to the lower extremities, and 15 (19.0%) to the upper extremities.

Analysis of longitudinal CT morphometric changes revealed that the degree of sarcopenic and adipose tissue decline varied significantly according to tumor localization. Patients with tumors of the trunk exhibited the most pronounced reductions across all muscle indices and visceral fat parameters.

**Fig. 4:**
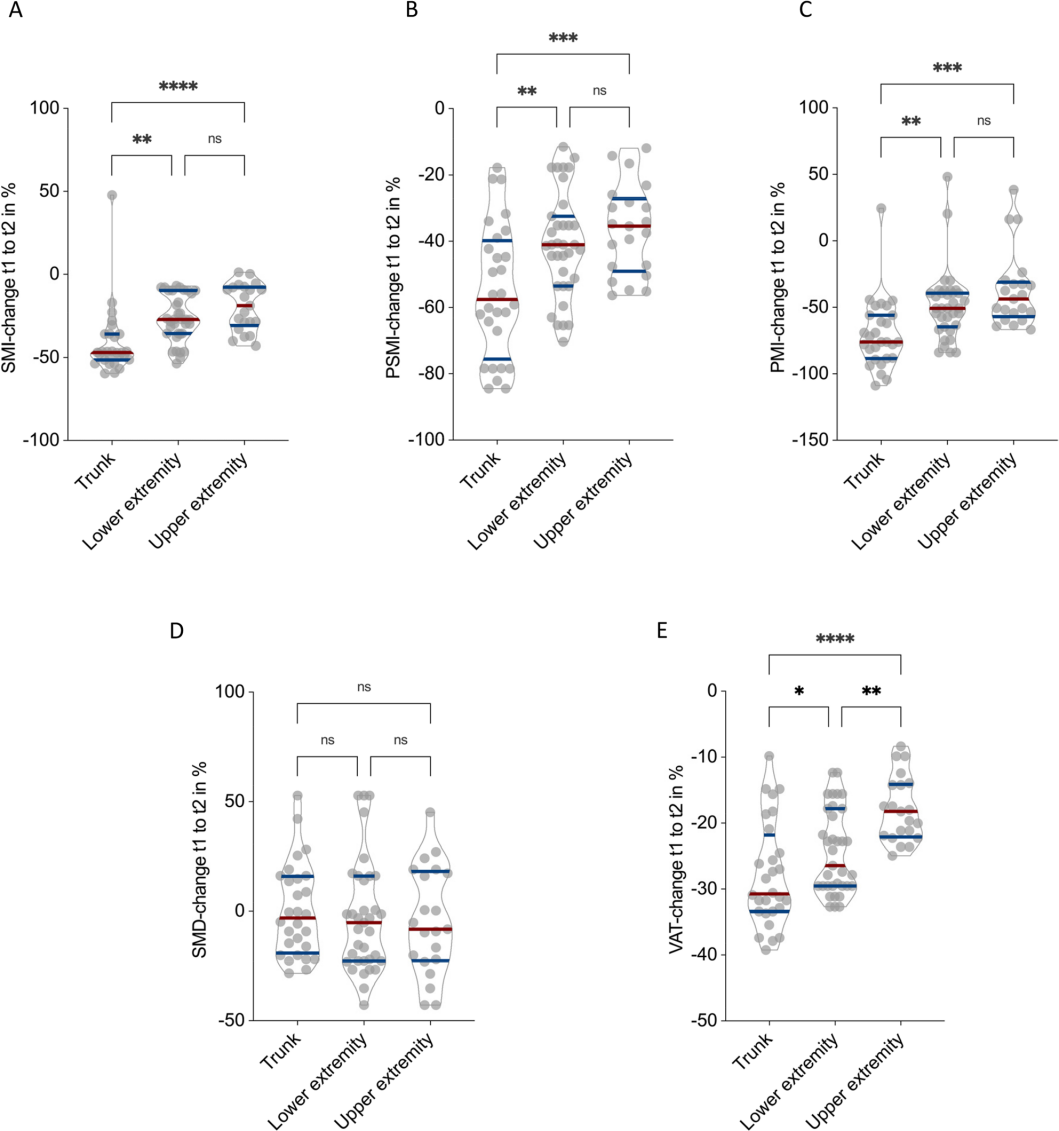
Influence of Tumor Localization on CT-Morphometric Decline Following Surgical Resection in Chondrosarcoma Patients. Relative percentage changes in SMI (**A**), PSMI (**B**), PMI (**C**), SMD (**D**), and VAT (**E**) stratified by tumor localization (trunk vs. lower extremity vs. upper extremity). Patients with trunk-localized chondrosarcomas exhibited the most pronounced postoperative loss of muscle and visceral fat (all p-values as indicated), while SMD remained stable across localizations. *p < 0.05, **p < 0.01 ***p < 0.001, ****p < 0.0001

SMI decreased by − 41.3 ± 20.4% in trunk-localized tumors, compared with − 26.5 ± 14.0% in lower-extremity tumors and − 19.5 ± 14.0% in upper-extremity tumors (*p* < 0.01 trunk vs. lower extremity; *p* < 0.0001 trunk vs. upper extremity; Fig. 4A). Similarly, PSMI declined by − 55.1 ± 20.0% in trunk tumors, − 40.9 ± 16.0% in lower extremities, and − 36.5 ± 13.7% in upper extremities (*p* < 0.01 trunk vs. lower; *p* < 0.001 trunk vs. upper; Fig. 4B).

PMI demonstrated the steepest relative decline, with a reduction of − 70.2 ± 26.1% in trunk tumors compared with − 48.4 ± 26.2% in lower-extremity and − 37.0 ± 28.5% in upper-extremity tumors (*p* < 0.01 trunk vs. lower; *p* < 0.001 trunk vs. upper; Fig. 4C). SMD remained stable across all localizations, with mean changes of − 0.3 ± 21.2% in trunk, − 2.7 ± 25.5% in lower extremities, and − 4.5 ± 24.4% in upper extremities (all *p* > 0.05; Fig. 4D).

VAT decreased by − 27.9 ± 24.4% in trunk-localized tumors, − 24.4 ± 6.4% in lower extremities, and − 17.8 ± 5.0% in upper extremities (*p* = 0.03 trunk vs. lower; *p* < 0.0001 trunk vs. upper; *p* < 0.01 lower vs. upper; Fig. 4E).

In summary, patients with trunk-localized chondrosarcomas experienced the most pronounced postoperative loss of SMI, PSMI, PMI, and VAT, indicating that tumor localization substantially affects the extent of surgical trauma, postoperative recovery, and systemic catabolic response following resection.

### High-grade chondrosarcomas exhibit accelerated postoperative sarcopenic decline

In addition to tumor localization, histological grade may further influence the extent of postoperative morphometric decline. To investigate whether more aggressive tumor biology is associated with a stronger catabolic response, subsequent analyses compared longitudinal changes in SMI, PSMI, PMI, SMD, and VAT across tumor grades.

**Fig. 5:**
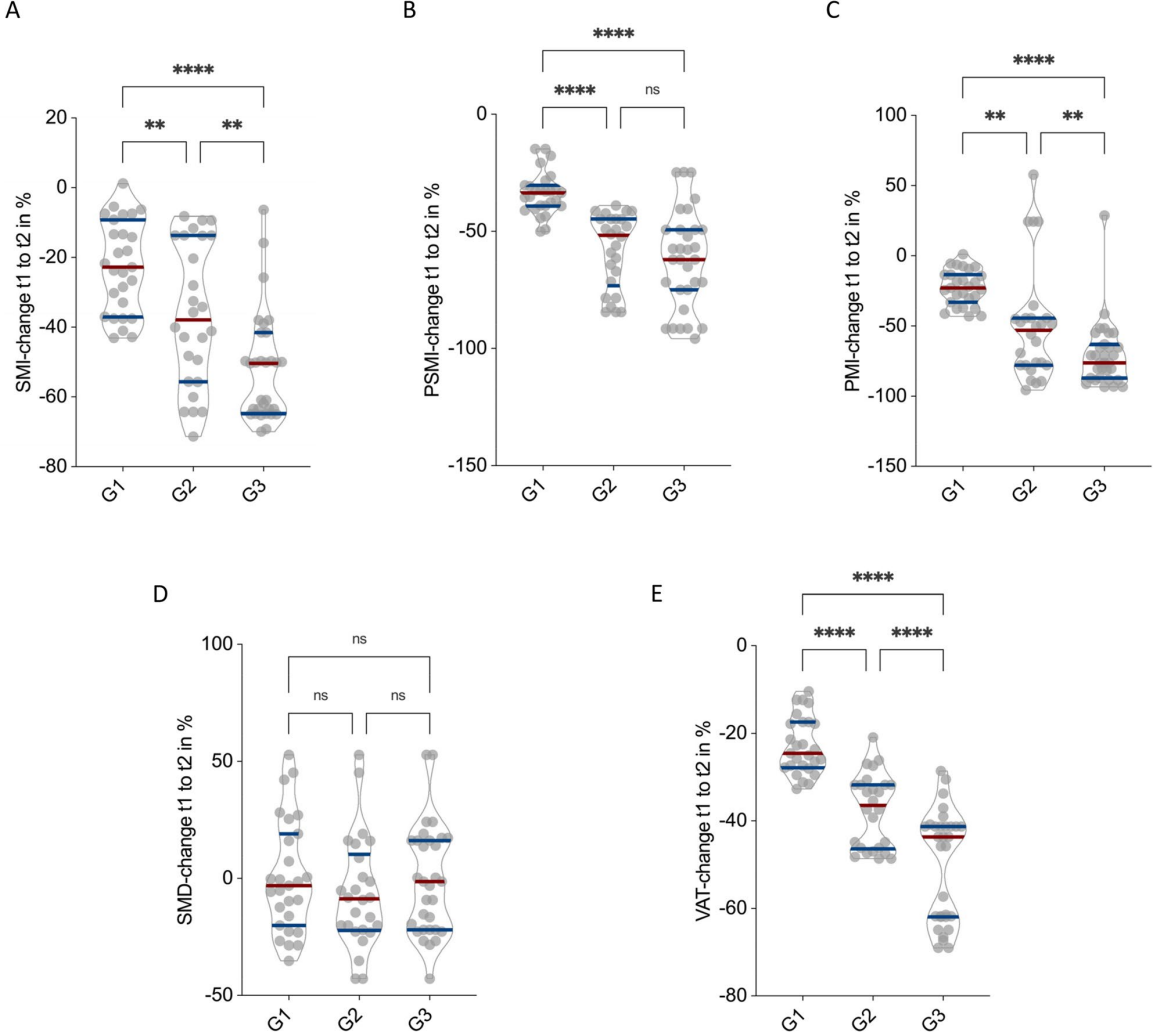
Impact of Tumor Grade on Postoperative CT-Morphometric Body Composition Decline in Chondrosarcoma Patients. Percentage changes in SMI (**A**), PSMI (**B**), PMI (**C**), SMD (**D**), and VAT (**E**) stratified by histological grade (G1, G2, G3). A stepwise increase in muscle and visceral fat loss was observed with increasing tumor grade, with G3 tumors demonstrating the steepest postoperative decline. SMD remained unaffected across grades. *p < 0.05, **p < 0.01 ***p < 0.001, ****p < 0.0001

Of the 79 surgically treated chondrosarcoma patients, 27 (34.2%) were classified as G1, 25 (31.6%) as G2, and 27 (34.2%) as G3 according to histopathological grading. To evaluate whether tumor grade influenced the extent of postoperative body composition decline, relative changes in CT morphometric parameters were compared across the three subgroups (Fig. 5).

A clear and stepwise increase in morphometric deterioration was observed with higher tumor grade. SMI declined by − 22.5 ± 13.2% in G1, − 36.3 ± 20.6% in G2, and − 52.1 ± 15.6% in G3 (*p* < 0.01 for G1 vs. G2; *p* < 0.0001 for G1 vs. G3; *p* < 0.01 for G2 vs. G3; Fig. 5A). PSMI decreased by − 33.7 ± 9.2% in G1, − 57.9 ± 15.9% in G2, and − 62.0 ± 20.9% in G3 (*p* < 0.0001 for G1 vs. G2 and G1 vs. G3; Fig. 5B).

PMI showed the steepest decline, with reductions of − 22.5 ± 12.6% in G1, − 49.7 ± 40.3% in G2, and − 70.6 ± 23.3% in G3 (*p* < 0.01 for G1 vs. G2; *p* < 0.0001 for G1 vs. G3; *p* < 0.01 for G2 vs. G3; Fig. 5C). SMD remained stable across all grades, with mean changes of 0.6 ± 24.1% in G1, − 6.5 ± 23.7% in G2, and − 0.8 ± 23.4% in G3 (all *p* > 0.05; Fig. 5D).

VAT decreased progressively with increasing tumor grade, from − 22.9 ± 6.5% in G1 to − 37.6 ± 8.4% in G2 and − 50.0 ± 12.6% in G3 (*p* < 0.0001 for all between-group comparisons; Fig. 5E).

In summary, higher tumor grade was strongly associated with a more pronounced postoperative decline in SMI, PSMI, PMI, and VAT, while SMD remained unaffected. This pattern suggests that aggressive tumor biology and the increased surgical burden in high-grade chondrosarcomas exacerbate postoperative catabolic processes and accelerate sarcopenic progression.

### Loss of SMI and VAT predicts decreased survival and functional deterioration

Building on these findings, we next examined whether postoperative morphometric decline translated into inferior survival outcomes and functional impairment. To ensure comparability, this analysis was restricted to the same R0-resected, N0M0 cohort, thereby eliminating confounding by residual disease or systemic therapy. The total number of events during the follow-up period was 39.

**Fig. 6:**
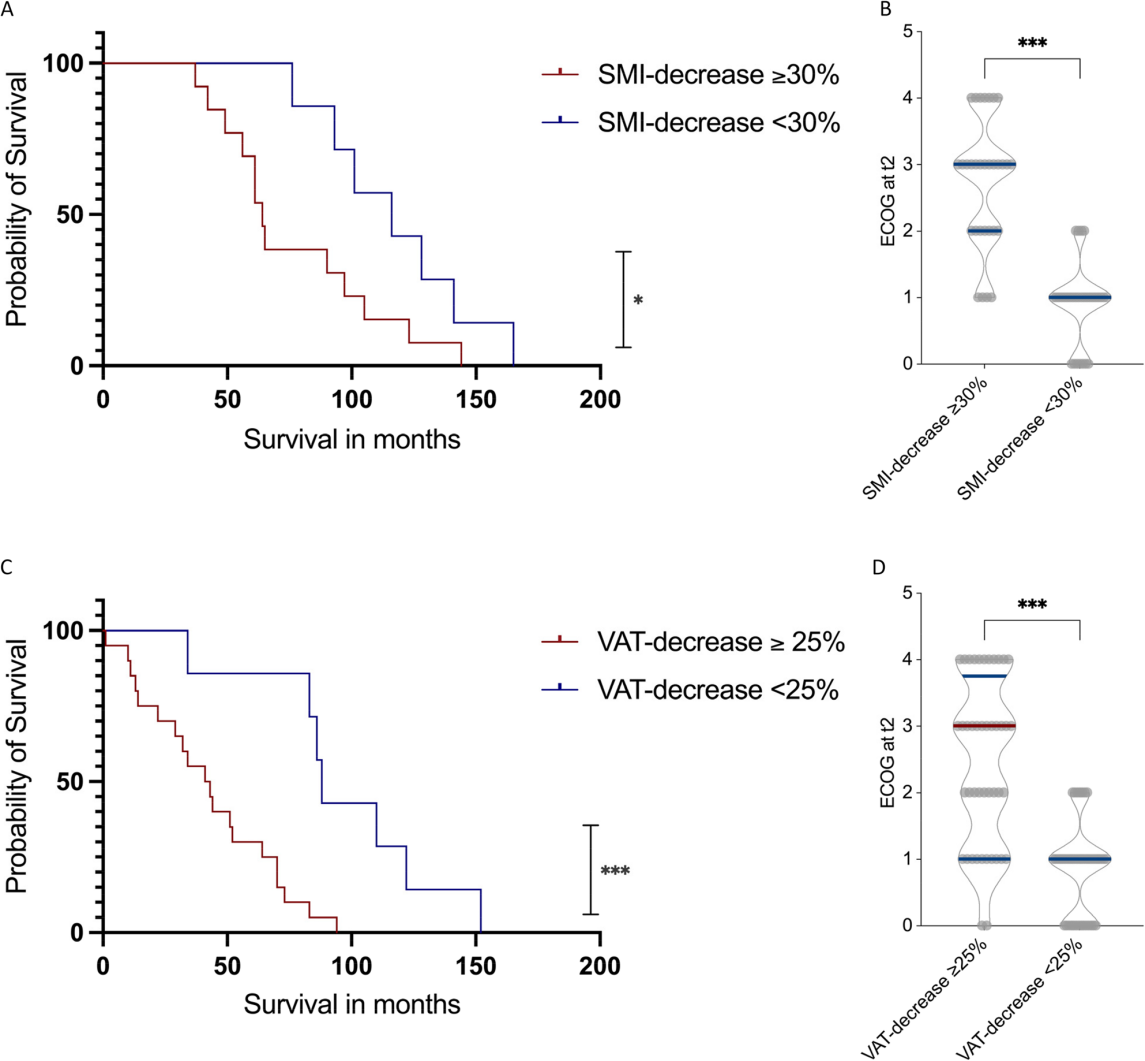
Prognostic Impact of Postoperative Loss of Skeletal Muscle and Visceral Fat in Chondrosarcoma Patients Following Surgical Resection. Kaplan–Meier analysis comparing overall survival in patients with ≥ 30% vs. <30% reduction in SMI (**A**), and ≥ 25% vs. <25% reduction in VAT (**C**). Patients with greater SMI or VAT decline exhibited significantly reduced survival (*p* = 0.02 and *p* < 0.01, respectively). Corresponding follow-up ECOG performance status scores (**B**, **D**) were significantly worse in patients with pronounced morphometric decline (both *p* < 0.001). *p < 0.05, **p < 0.01 ***p < 0.001, ****p < 0.0001

Kaplan–Meier survival analysis demonstrated a significantly reduced overall survival in patients with a pronounced postoperative decline in SMI and VAT. Patients with an SMI decrease ≥ 30% had a median OS of 64 months, compared with 116 months in those with an SMI decrease < 30% (*p* = 0.02; Fig. 6A). In parallel, ECOG performance status at t2 was markedly worse in the SMI ≥ 30% subgroup, with a median ECOG of 3.0 (range 1.0) compared with 1.0 (range 0.8) in the < 30% loss group (*p* < 0.001; Fig. 6B).

Similarly, patients with a VAT decrease ≥ 25% exhibited a median OS of 42 months, whereas those with < 25% VAT loss had a median OS of 88 months (*p* < 0.01; Fig. 6C). ECOG performance status at t2 was likewise significantly impaired in patients with marked VAT loss (median 3.0 ,range 1.5 vs. 1.0, range 0.8, *p* < 0.001; Fig. 6D).

To further substantiate these findings and evaluate the independent prognostic contribution of SMI and VAT decline, univariate and multivariate Cox proportional hazards regression analyses were performed (Tables 2 and 3).

**Table 2:** Univariate cox hazard regression model

Predictor	Hazard Ratio	95%-Confidence Interval	*p*-Value
Age	1.027	1.005–1.09	< 0.01**
Sex	0.81	0.32–1.89	0.511
Tumor diameter	1.34	1.02–1.55	< 0.01**
ECOG	1.31	1.21–1.81	< 0.001***
Tumor localization	1.33	1.11–2.51	0.01*
Grading	1.41	1.19–2.50	0.02*
SMI-decrease	1.49	1.10–2.15	0.02*
VAT-decrease	1.44	1.22–2.31	0.01*
Sarcopenia pre-surgery	1.41	1.31–2.40	< 0.001***

Illustrated are the results of the univariate cox hazard regression model

*Abbreviations:* *ECOG* Eastern Cooperative Oncology Group, *SMI* Skeletal Muscle Index, *VAT* Visceral adipose tissue

*p < 0.05, **p < 0.01 ***p < 0.001

**Table 3:** Multivariate cox hazard regression model

Predictor	Hazard Ratio	95%-Confidence Interval	*p*-Value
Age	1.011	0.88–1.09	0.33
Sex	0.79	0.31–1.91	0.44
Tumor diameter	1.29	1.02–1.51	0.03*
ECOG	1.21	1.19–1.75	0.02*
Tumor localization	1.31	1.11–2.55	0.02*
Grading	1.31	1.17–2.51	0.02*
SMI-decrease	1.44	1.10–2.33	0.02*
VAT-decrease	1.40	1.20–2.41	0.01*
Sarcopenia pre-surgery	1.31	1.19–2.45	< 0.01**

Illustrated are the results of the multivariate cox hazard regression model

*Abbreviations:* *ECOG* Eastern Cooperative Oncology Group, *SMI*: Skeletal Muscle Index, *VAT* Visceral adipose tissue

*p < 0.05, **p < 0.01

In univariate analysis, age, tumor diameter, ECOG, tumor localization, grading, SMI decrease ≥ 30%, and VAT decrease ≥ 25% were significantly associated with decreased survival. In multivariate analysis, both SMI decrease ≥ 30% (HR 1.44, 95% CI 1.10–2.33, *p* = 0.02) and VAT decrease ≥ 25% (HR 1.40, 95% CI 1.20–2.41, *p* = 0.01) remained independent predictors of worse OS after adjustment for age, sex, tumor diameter, ECOG, tumor localization, and grading.

Taken together, these results demonstrate that postoperative losses of SMI ≥ 30% and VAT ≥ 25% independently predict inferior survival and functional outcomes in R0-resected N0M0 chondrosarcoma patients. The integration of CT-based morphometric analysis thus provides a valuable prognostic tool to identify surgically treated patients at risk for unfavorable postoperative trajectories.

### Preoperative sarcopenia predicts inferior survival and increased postoperative morbidity

**Fig. 7:**
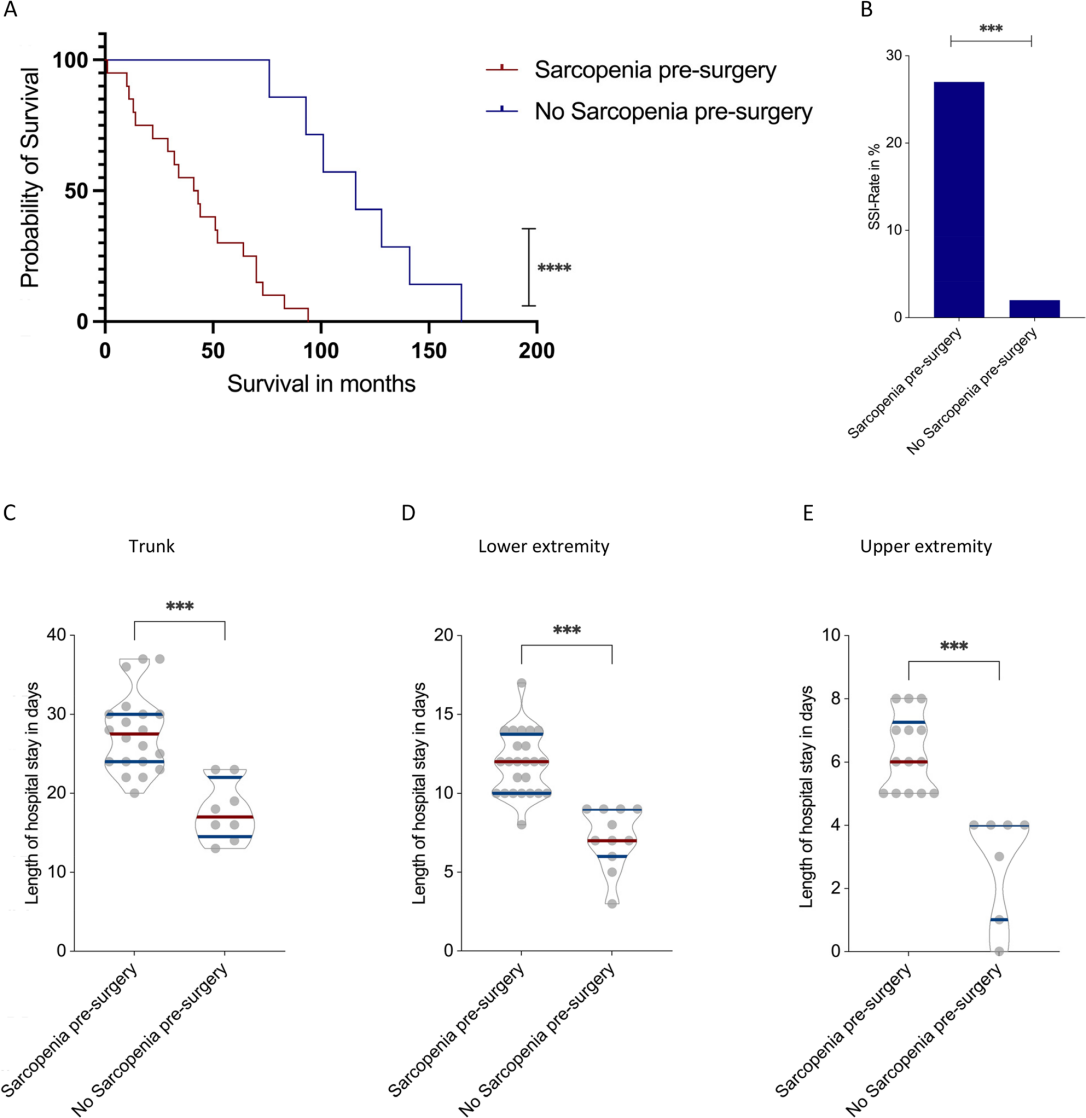
Effect of Preoperative Sarcopenia on Survival and Postoperative Morbidity in Chondrosarcoma Patients. Comparisons between sarcopenic and non-sarcopenic patients at baseline regarding overall survival (**A**), rate of surgical site infection (SSI) (**B**), and length of postoperative hospitalization stratified by tumor localization (**C**–**E**). Sarcopenia was associated with markedly reduced survival (*p* < 0.0001), higher SSI rates (*p* < 0.001), and prolonged hospitalization across all anatomical sites (all *p* < 0.001). *p < 0.05, **p < 0.01 ***p < 0.001, ****p < 0.0001

Given that postoperative sarcopenic progression adversely affected long-term survival and function, we next investigated whether sarcopenia present before surgery already predisposed patients to inferior outcomes. This analysis focused on preoperative SMI values to determine whether baseline muscle depletion influences survival, postoperative complications, and recovery trajectories following R0 resection. Sarcopenia was defined using commonly applied sex-specific SMI thresholds (< 52.4 cm²/m² for men and < 38.5 cm²/m² for women) originally derived by Prado et al. using optimal stratification of CT-derived skeletal muscle index in a large oncologic cohort [14]. These thresholds have subsequently been widely adopted in CT-based body composition research across different malignancies when disease-specific cut-offs are unavailable. Importantly, several studies investigating body composition in sarcoma populations have applied the same Prado thresholds to define sarcopenia and demonstrated their prognostic relevance for survival and postoperative outcomes, supporting their applicability in musculoskeletal oncology cohorts [15, 16]. Kaplan–Meier survival analysis revealed a markedly reduced overall survival in sarcopenic patients. The median survival was 42 months in the sarcopenic group compared with 116 months in non-sarcopenic patients (*p* < 0.0001; Fig. 7A). In univariate Cox regression analysis, preoperative sarcopenia was significantly associated with decreased survival (HR 1.41, 95% CI 1.31–2.40, *p* < 0.001), and this association remained significant in the multivariate model after adjustment for age, sex, tumor size, ECOG, tumor localization, and grade (HR 1.31, 95% CI 1.19–2.45, *p* < 0.01), confirming preoperative sarcopenia as an independent predictor of poor survival.

Sarcopenia was also associated with a substantially higher postoperative morbidity. The rate of SSI was significantly elevated in sarcopenic patients (27%) compared with only 2% in non-sarcopenic patients (*p* < 0.001; Fig. 7B).

When analyzing postoperative hospital stay by tumor localization, sarcopenic patients consistently demonstrated longer recovery durations across all anatomic sites, even after excluding cases with vacuum-assisted wound closure as potential confounders. In trunk-localized tumors, mean hospitalization was 27.7 ± 5.0 days in sarcopenic patients versus 17.8 ± 3.8 days in non-sarcopenic patients (*p* < 0.001; Fig. 7C). In lower-extremity tumors, hospital stay averaged 11.9 ± 2.0 days compared with 7.2 ± 1.9 days (*p* < 0.001; Fig. 7D), while for upper-extremity tumors, hospitalization was 6.3 ± 1.2 days versus 1.2 ± 1.7 days (*p* < 0.001; Fig. 7E).

In summary, preoperative sarcopenia was independently associated with worse survival, higher postoperative complication rates, and prolonged hospitalization following complete (R0) resection of N0M0 chondrosarcomas. These findings highlight the clinical significance of preoperative muscle depletion as a modifiable prognostic factor and underscore the importance of early identification and prehabilitation in surgically treated chondrosarcoma patients.

## Discussion

This study demonstrates that in R0-resected, non-metastatic chondrosarcoma, body composition parameters derived from routine CT imaging provide valuable prognostic and clinical information. Across the cohort, a consistent decline in skeletal muscle and visceral adipose tissue was observed over the disease course, whereas muscle density remained largely stable. This decline was not uniform across all patients: those with trunk-localized and high-grade tumors experienced the most pronounced losses. Importantly, postoperative decreases in SMI of ≥ 30% and VAT of ≥ 25% independently predicted inferior overall survival and worse functional outcomes, even after adjustment for established clinical and pathological prognostic factors. Likewise, preoperative sarcopenia identified patients at increased risk for postoperative complications, prolonged hospitalization, and decreased long-term survival.

These findings indicate that both tumor- and host-related factors contribute to postoperative body composition changes in chondrosarcoma. Tumors located in the trunk or lower extremities often necessitate more extensive surgical exposure, complex reconstruction, and prolonged postoperative immobilization, which together accelerate muscle and fat loss [17, 18]. High-grade tumors additionally impose a stronger systemic inflammatory and metabolic burden, further promoting catabolic depletion [19–21]. Preoperative sarcopenia likely reflects reduced physiological reserve, consistent with its association with increased surgical site infection and extended hospitalization. In contrast, pronounced postoperative declines in SMI and VAT represent ongoing treatment-related deterioration that influences both survival and functional recovery. The relative stability of muscle density suggests that these early changes predominantly reflect loss of muscle quantity rather than quality.

The present results are consistent with previous longitudinal sarcoma analyses, which likewise demonstrated progressive declines in SMI, PSMI, PMI, and VAT over the disease course. In myxofibrosarcoma, muscle and fat loss were linked to shorter survival and higher postoperative morbidity, while muscle density remained largely stable [15]. Similarly, in osteosarcoma, baseline sarcopenia and marked postoperative SMI decline predicted poorer survival and impaired functional status, particularly in patients with elevated systemic inflammation [22]. However, in both of these cohorts, systemic therapies such as chemotherapy and radiotherapy were integral components of treatment and are known to exert independent effects on muscle metabolism and body composition.

In contrast, the present chondrosarcoma cohort was treated exclusively with surgical resection, without chemotherapy or radiotherapy, and consisted solely of R0-resected, non-metastatic (N0M0) cases. This design isolates the impact of surgery itself on postoperative morphometric decline, eliminating confounding effects from systemic treatment-related cachexia. Within this purely surgically managed population, the extent of muscle and visceral fat loss was driven by tumor localization and histologic grade, highlighting the role of surgical burden and tumor biology in shaping postoperative trajectories. Moreover, this study identifies clear and clinically applicable thresholds of postoperative decline (SMI ≥ 30% and VAT ≥ 25%) as independent predictors of survival and functional outcome, thereby providing prognostic markers that are directly relevant to surgical decision-making.

In line with this, preoperative identification of sarcopenic patients through regular staging CT morphometry allows for tailored perioperative strategies, including nutritional optimization, targeted resistance training, and enhanced postoperative rehabilitation [23, 24]. In complex axial resections or high-grade tumors, these measures may be particularly important to mitigate the pronounced catabolic effects observed in this subgroup. Early postoperative monitoring of SMI and VAT, when follow-up CT scans are routinely obtained for tumor surveillance, may help identify patients at risk who could benefit from intensified supportive interventions. Furthermore, the observed association between preoperative sarcopenia and surgical site infections highlights the need for stringent perioperative infection prevention, potentially including negative-pressure closed incisional wound therapy, optimized antibiotic protocols, early nutritional supplementation as well as prehabilitation. From a health systems perspective, recognizing sarcopenic patients preoperatively may facilitate more individualized planning of hospital stays, rehabilitation needs, and postoperative follow-up intensity.

Several methodological aspects support the robustness of this analysis. The study cohort consisted exclusively of R0-resected, non-metastatic (N0M0) chondrosarcoma patients, which helped to reduce potential confounding by residual disease or systemic therapy. Additionally, the availability of two CT time points separated by at least twelve months allowed for a meaningful longitudinal assessment of body composition changes.

Nevertheless, several limitations must be acknowledged. The retrospective, single-center design inherently carries the risk of selection bias. Furthermore, the requirement for two evaluable CT scans for longitudinal morphometric analysis may have excluded patients with early mortality or rapid disease progression who did not undergo follow-up imaging. This introduces a potential survivorship bias and may lead to an underrepresentation of patients with particularly aggressive disease courses. Given the relatively limited sample size and number of events, inclusion of multiple covariates in the multivariable Cox models may introduce a risk of model overfitting. Therefore, the results should be interpreted cautiously and require confirmation in larger multicenter cohorts. Factors such as nutritional intake, systemic inflammation, corticosteroid exposure, and physical activity could not be controlled for and may have influenced body composition changes. While L3-level measurements are well validated as surrogates for whole-body composition, they may not capture localized muscle wasting in certain anatomical regions. Additionally, although CT acquisition was standardized, minor variations in technical settings could affect SMD readings. Additionally, because these thresholds were derived within the present cohort using ROC analysis, they should be interpreted as exploratory markers and require validation in independent patient populations. Although penalized regression or model reduction approaches may reduce overfitting risk in smaller datasets, the multivariable models in the present study were intentionally constructed using predefined clinically established prognostic variables to preserve clinical interpretability and comparability with prior sarcoma outcome studies. Nonetheless, the results should be interpreted with caution. Finally, by focusing on surgically treated, non-metastatic patients without chemotherapy or radiotherapy, the generalizability of these findings to patients undergoing multimodal treatment remains to be verified.

Future research should aim to validate these findings in multicenter, prospective settings, ideally incorporating patients across the entire spectrum of chondrosarcoma biology and treatment modalities. Prospective interventional studies could explore whether structured prehabilitation or targeted nutritional and physical therapy programs can attenuate postoperative morphometric decline and improve functional outcomes. Advances in automated CT morphometry, particularly through artificial intelligence-assisted L3 segmentation, may facilitate integration of body composition assessment into standard radiologic workflows, enabling real-time risk stratification. Furthermore, combining CT-based metrics with circulating biomarkers of inflammation and metabolism may refine predictive models and enhance individualized perioperative care.

In conclusion, this study demonstrates that both preoperative sarcopenia and postoperative declines in SMI and VAT are independent predictors of reduced survival, impaired functional recovery, and increased postoperative morbidity in surgically treated, non-metastatic chondrosarcoma patients. The extent of morphometric loss is particularly pronounced in trunk-localized and high-grade tumors. CT-based morphometric analysis, readily available from routine imaging, may therefore provide a pragmatic adjunct for risk stratification and perioperative assessment. However, the identified ROC-derived thresholds should be considered exploratory and require external validation in larger multicenter cohorts before clinical implementation. Early identification and management of sarcopenia may represent a potentially modifiable avenue to improve oncologic and functional outcomes in chondrosarcoma surgery.

## Data Availability

All data is provided within the manuscript.
